# Diagnosis of Rare Association of Orthotopic Multicystic Dysplasia with Crossed Fused Renal Ectopia

**DOI:** 10.1155/2014/140850

**Published:** 2014-04-15

**Authors:** Linnan Tang, June Koshy, Melissa R. Spevak, Jane E. Benson, Thangamadhan Bosemani

**Affiliations:** Division of Pediatric Radiology, Russell H. Morgan Department of Radiology and Radiological Science, The Johns Hopkins University School of Medicine, Charlotte R. Bloomberg Children's Center, Sheikh Zayed Tower, Room 4174, 1800 Orleans Street, Baltimore, MD 21287-0842, USA

## Abstract

Orthotopic multicystic dysplastic kidney with crossed fused ectopia is a rare congenital anomaly. This congenital anomaly may give an appearance of a solitary kidney morphology during the initial imaging evaluation. A solitary kidney should be carefully evaluated for the presence of duplication, horseshoe configuration, or crossed renal ectopy. Vesicoureteral reflux is a common finding associated with a multicystic dysplastic kidney. We present an infant with an orthotopic multicystic dysplastic kidney and an inferiorly placed crossed fused ectopic kidney. The presence of a complex congenital anomaly may warrant further evaluation with cross-sectional imaging to depict the anatomy and structure.

## 1. Introduction

Crossed renal ectopia (CRE) is a congenital anomaly where the ectopic kidney crosses the midline with the ureter at the ureterovesical junction (UVJ) remaining in a contralateral position. CRE has been reported in 1 : 2000 to 1 : 7000 autopsies [1]. Fusion of the ectopic kidney to the contralateral orthotopic site is present in 80–90% of cases [2, 3]. Multicystic Dysplastic Kidney (MCDK) in a duplex kidney is a rare presentation. There is only one reported case in the literature of an orthotopic MCDK and fused CRE [4] to the best of our knowledge. We report this rare association of an orthotopic right MCDK with a left fused CRE, where the diagnosis required a multimodality imaging approach.

## 2. Case Report

**Figure 1:**
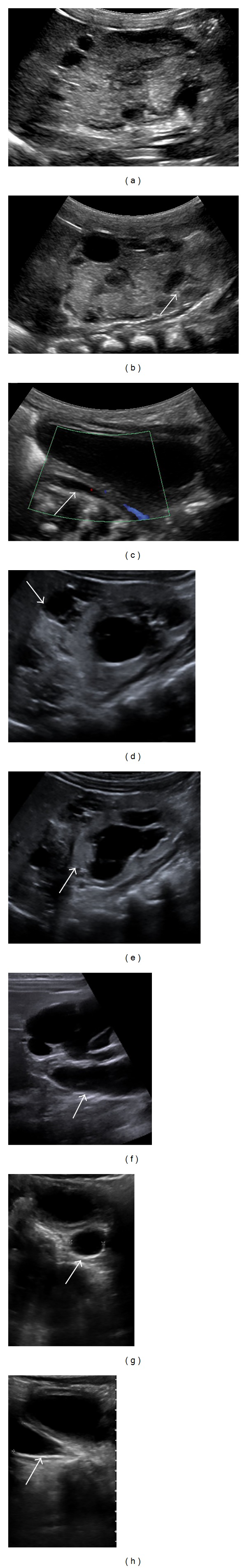
Abdomen US, day 0 of life (a)–(c). (a) and (b) sagittal images, solitary right kidney: cysts in the upper pole, increased renal parenchymal echogenicity, and mild dilatation of the renal pelvis (arrow); (c) right parasagittal image, bladder: mild dilatation of the right ureter extending to the ureterovesical junction (arrow). Abdomen US, 6 months of age (d)–(h). (d) sagittal image, right flank: noncommunicating cysts with no intervening renal parenchyma consistent with a right multicystic dysplastic kidney (arrow) and moderate hydronephrosis of the inferior crossed fused left kidney; (e) sagittal image shows increased renal parenchymal echogenicity of the crossed fused left kidney with relative preservation of parenchymal thickness (arrow); (f) sagittal image shows moderate dilatation of the proximal left ureter to the right of midline consistent with crossed fused ectopy; (g) transverse image, bladder: dilated distal left ureter confirms the crossed fused left renal ectopy; (h) sagittal image, bladder: dilated tapered atretic right ureter.

A 36-week gestation male infant was born with dysmorphic facial features, right ear microtia, bilateral external auditory canal stenosis, and a single umbilical artery. Screening abdomen ultrasound (US) at day 0 of life demonstrated a solitary kidney on the right with multiple noncommunicating cysts in the upper pole, giving the appearance of a segmental MCDK. The US showed renal pelvis prominence and a mildly dilated ureter extending to the UVJ (Figure 1). The left renal fossa was empty. Echocardiographic evaluation revealed atrial and ventricular septal defects. The infant was discharged home on prophylactic antibiotics and was scheduled for follow-up in an outpatient clinic.

**Figure 2:**
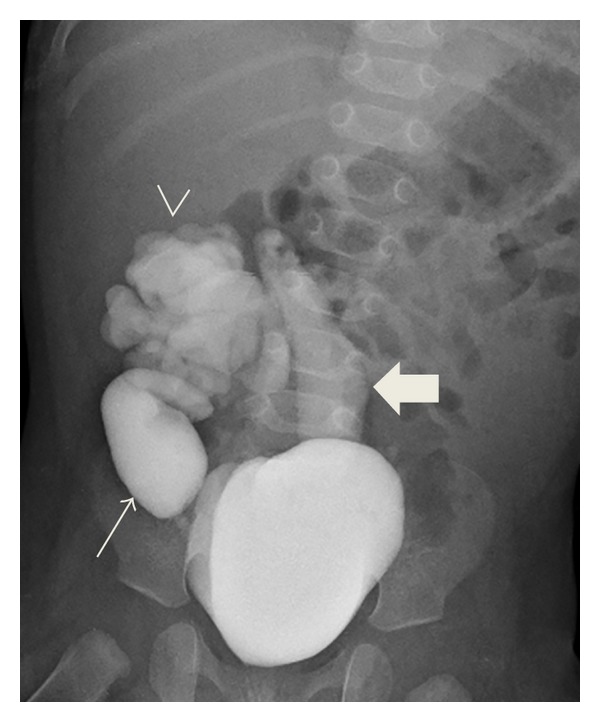
VCUG, 6 months of age: dilated atretic right ureter with high grade vesicoureteral reflux (arrow), high grade vesicoureteral reflux into tortuous left ureter that lies to the left of midline (block arrow), and the dilated left collecting system with blunting of the calyces to the right of midline (arrowhead), suggestive of left crossed fused renal ectopy.

**Figure 3:**
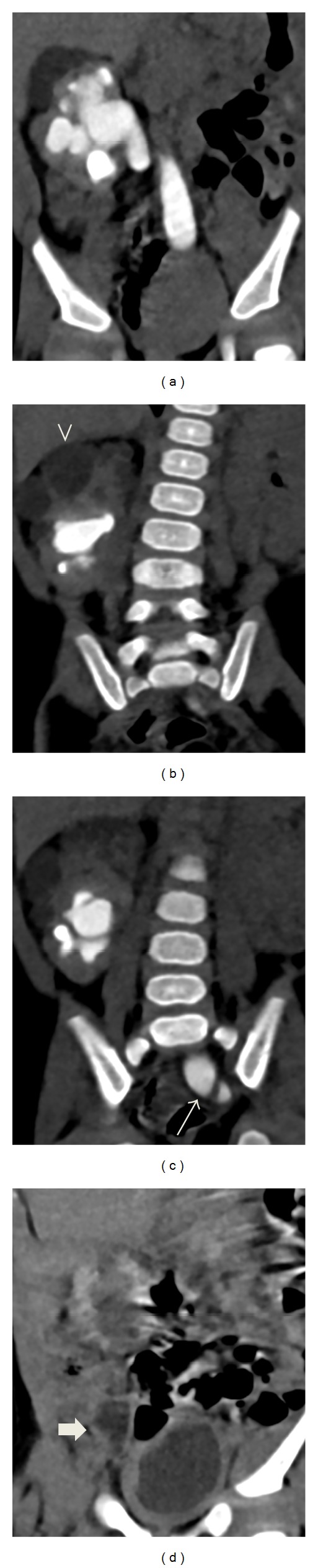
Contrast-enhanced abdomen CT, 6 months of age. (a) moderate hydronephrosis in the left crossed fused renal ectopic kidney; (b) noncommunicating cysts representing the orthotopic right multicystic dysplastic kidney in the superior aspect (arrowhead); (c) dilated tortuous left ureter at the left ureterovesical junction (arrow); (d) nonopacified right distal atretic ureter is not opacified (block arrow) confirming the absence of communication with the nonfunctioning orthotopic right multicystic dysplastic kidney.

**Figure 4:**
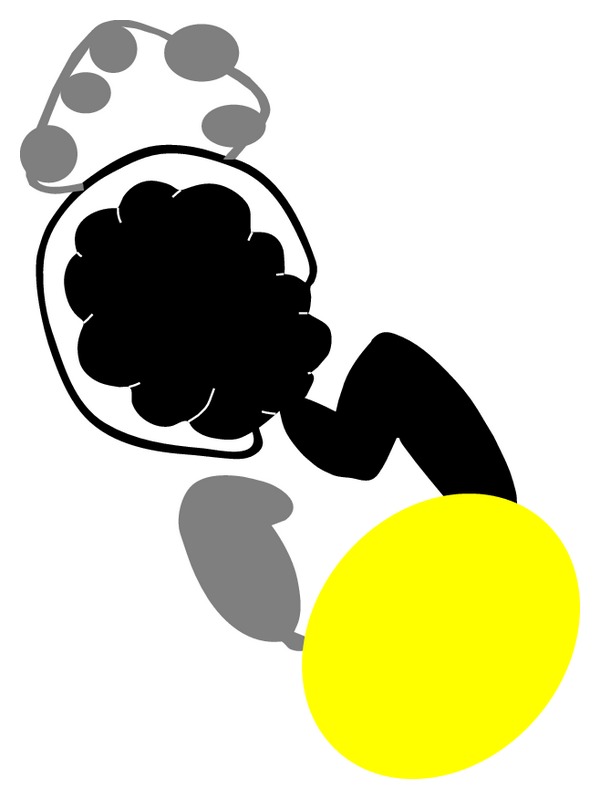
Schematic representation of the anatomic structures: Moderate hydroureteronephrosis (black) of left crossed fused ectopic kidney; atretic dilated right ureter (gray), and noncommunicating cysts in multicystic dysplastic right kidney (gray) and urinary bladder (yellow).

At 6 months of age, the infant presented with fever, lethargy, and pyuria. Renal US demonstrated moderate hydronephrosis in the lower pole moiety of the solitary kidney on the right, which was previously a mildly prominent renal pelvis. In addition, moderate bilateral distal ureteric dilatation was present; the finding on the left was new however. The cysts in the upper pole right MCDK had mildly involuted in size in comparison to the prior examination. The left distal ureteric dilatation raised the suspicion for CRE and inferior fusion of an ectopic left kidney to the orthotopic right MCDK. Voiding cystourethrogram (VCUG) showed marked vesicoureteral reflux (VUR) in the left ureter which crosses over to the right of midline, consistent with CRE and marked VUR in the distal right atretic ureter (Figure 2). The patient's fever did not respond to antibiotics and concern for an intra-abdominal source of sepsis was raised and hence a contrast-enhanced computed tomography (CT) of the abdomen and pelvis was obtained. CT showed a right MCDK with no parenchymal enhancement. Delayed images showed absence of opacification of the dilated distal right ureter, confirming its discontinuity with the upper tract and representing an atretic segment. Continuity of the left ureter with the inferior renal unit on the right confirmed the diagnosis of a left CRE (Figure 3). A schematic representation of the anatomy is shown (Figure 4). No acute intra-abdominal process was identified.

He remained in the hospital for a week with an indwelling urinary catheter to facilitate free drainage of urine. His fever had subsequently resolved. The patient was discharged home on prophylactic antibiotics for the high grade VUR. A surgical plan was being formulated to treat the VUR.

## 3. Discussion

MCDK in a duplex kidney is a rare presentation but well known [5]. While it can occur on either side, upper pole involvement predominates. There have been several previously reported cases of MCDK in the ectopic kidney, both fused and unfused [6–9]. MCDK is often associated with contralateral VUR and ureteropelvic junction obstruction (UPJO) [10]. Here we demonstrate a rare presentation of MCDK in the orthotopic kidney with crossed fused renal ectopy. The initial imaging presentation demonstrated a solitary kidney morphology with unilateral ureteric dilatation. Follow-up imaging showed bilateral distal ureteric dilatation requiring a multimodality imaging approach to establish the correct diagnosis.

Embryological development is complex, with sequential formation and degeneration of two protokidneys, before a ureteric bud from the bladder interacts with the ipsilateral renal anlage to form the collecting system and renal parenchyma, respectively. A faulty ureteric bud or renal anlage may lead to failed induction and fusion, which theoretically explains the outcome of MCDK. The “ascent” of the kidneys in the fetal retroperitoneum is mainly due to the lengthening of the trunk: the bladder and kidneys “grow apart”. Anomalous migration that results in ectopy (crossed or pelvic) might be explained by pressure on or distortion of the ureteric bud by the growing surrounding fetal tissue so that it induces renal formation on the contralateral side. Tethering or blocking of the ureteric bud or developing kidney by an umbilical artery, so that it is deflected, is an alternative explanation [11].

CRE is typically asymptomatic; however it can be associated with abdominal or flank pain, palpable mass, hematuria, dysuria, or urinary tract infections. There may also be associated nephrolithiasis, UPJO, hydronephrosis, VUR, and ectopic ureteroceles. Patients may also present with multiple congenital anomalies [1, 3, 12]. In our patient, the presence of a single umbilical artery, microtia, external auditory canal stenosis, and cardiac malformations prompted the abdomen US in particular to look for renal anomalies.

US evaluation in day 0 of life underestimated the hydronephrosis and ureteric dilatation, likely due to dehydration of the newborn infant [10]. The left ureteric dilatation in the subsequent US examination raised the suspicion of left CRE. VCUG is the test of choice to determine the presence of VUR. Cross-sectional imaging is often helpful to demonstrate the anatomy and provide functional information of such complex anomalies. Magnetic resonance Imaging (MRI) would be the ideal test due to the absence of radiation, but patient condition may dictate the choice. In an acute setting of infection or sepsis, CT may be considered. A DMSA scan is a radionuclide scan that may help in assessing the renal morphology and structure. The parenchymal enhancement demonstrated on the CT scan in our patient was considered adequate in revealing renal function and hence nuclear imaging was not performed.

We report here only the second recorded case of CRE with inferior fusion to an orthotopic MCDK. US, VCUG, and possibly CT/MRI may be required in the diagnostic pathway and clinical management of these children. The embryogenesis, clinical phenotype, and the physiologic state of the infant at the time of imaging are important considerations to make to arrive at the diagnosis.
